# A randomised double-blind placebo-controlled 12- week feasibility trial of methotrexate added to treatment as usual in early schizophrenia: study protocol for a randomised controlled trial

**DOI:** 10.1186/1745-6215-16-9

**Published:** 2015-01-07

**Authors:** Imran B Chaudhry, Nusrat Husain, Raza ur Rahman, Mohammed Omair Husain, Mohammed M Hamirani, Ajmal Kazmi, Shakeel Baig, Peter M Haddad, Maya H Buch, Inti Qureshi, Nasir Mehmood, Tayyeba Kiran, Bo Fu, Salahuddin Afsar, Bill Deakin

**Affiliations:** University of Manchester & Lancashire Care Early Intervention Service, Manchester, UK; Dow University of Health Sciences, Karachi, Pakistan; Southwest London and St George’s NHS Trust, London, UK; Abbassi Shaheed Hospital, Karachi, Pakistan; Pakistan Institute of Leaning and Living, Karachi, Pakistan; Baqai University, Karachi, Pakistan; University of Manchester & Greater Manchester West Mental Health NHS Foundation Trust, Manchester, UK; University of Leeds, Leeds, UK; Mersey Care NHS Trust, Liverpool, UK; University of Manchester, Manchester, UK

## Abstract

**Background:**

Methotrexate is a commonly used anti-inflammatory and immunosuppressive drug. There is growing evidence that inflammatory processes are involved in the pathogenesis of schizophrenia. In our recent randomised double-blind placebo-controlled clinical trial in Pakistan and Brazil, the addition of minocycline (antibiotic and anti-inflammatory drug) for 1 year to treatment as usual reduced negative symptoms and improved some cognitive measures. A meta-analysis of cytokine changes in the peripheral blood has identified IL-2, IFN-gamma, TNF-alpha and soluble IL-2 receptor as trait markers of schizophrenia because their levels were elevated during acute exacerbations and reduced in remission. This suggests immune activation and an inflammatory syndrome in schizophrenia. Based on the evidence of the strong anti-inflammatory properties of methotrexate, we propose that low-dose methotrexate may be an effective therapy in early schizophrenia.

**Methods/Design:**

This is a double-blind placebo-controlled study of methotrexate added to treatment as usual for patients suffering from schizophrenia, schizoaffective disorder, psychosis not otherwise specified or schizophreniform disorder. This will be with 72 patients, 36 in each arm over 3 months. There will be screening, randomisation and follow-up visits. Full clinical assessments will be carried out at baseline, 2, 4, 8 and 12 weeks. Social and cognitive assessments will be carried out at baseline and 12 weeks. Methotrexate will be given at a dose of 10 mgs orally once a week for a 3-month period.

**Discussion:**

Evidence suggests inflammatory processes are involved in the pathogenesis of schizophrenia and anti-inflammatory treatments have shown to have some beneficial effects. Methotrexate is a known immunosuppressant and anti-inflammatory drug. The aim of this study is to establish the degree of improvement in positive and negative symptoms, as well as cognitive functioning with the addition of methotrexate to treatment as usual.

ClinicalTrials.gov identifier: NCT02074319 (24 February 2014).

## Background

Methotrexate (MXTis a commonly used anti-inflammatory and immunosuppressive drug, originally developed and continuing to be used for chemotherapy either alone or in combination with other agents. It is used as a treatment for some autoimmune diseases, including arthritis, psoriasis, psoriatic arthritis, lupus and Crohn's disease. Low-dose MXT has been used for treatment of rheumatoid arthritis for almost 50 years, is very effective and is recognised as treatment of choice and first-line therapy for many patients [1]. MXT is thought to affect cancer and rheumatoid arthritis by two different pathways. For cancer, MXT competitively inhibits dihydrofolate reductase (DHFR), an enzyme that participates in the tetrahydrofolate synthesis [2]. However, in rheumatoid arthritis it most likely acts by the inhibition of enzymes involved in purine metabolism or by inhibition of T cell activation [3].

There has been interest in using MXT in other conditions where excessive immune activation and inflammatory mediators are believed to be involved in the pathogenesis. For example, Gong *et al*. [4] showed that in cardiac failure patients, low-dose MXT has significant anti-inflammatory effects and improved functional outcomes and quality of life. In an animal model of cardiac failure Zhang *et al*. [5] showed that MXT has the ability to regulate inflammatory responses and improve cardiac function, hence contributing to prevent the development of post-myocarditis dilated cardiomyopathy.

There is growing evidence that inflammatory processes are involved in the pathogenesis of schizophrenia [6]. In our recent randomised double-blind placebo-controlled clinical trial in Pakistan and Brazil, funded by the Stanley Medical Research Institute (SMRI), the addition of minocycline (an antibiotic and anti-inflammatory drug) for 1 year to treatment as usual (TAU) reduced negative symptoms and improved some cognitive measures [7]. Several studies have noted increase in peripheral inflammatory cytokines, such as IL-1, IL-2, IL-6, and TNF-alpha [8]. In the brain, cytokines seem to be involved in regulating the action of several neurotransmitters, such as serotonin, noradrenalin, dopamine, and glutamate. The interaction of cytokines with dopamine and glutamate seems to be especially relevant to the pathophysiology of schizophrenia [9].

A meta-analysis of cytokine changes in the peripheral blood has identified IL-12, IFN-gamma, TNF-alpha, and soluble IL-2 receptor (sIL-2R) as trait markers of schizophrenia because their levels were elevated during acute exacerbations and remission [10]. An extensive meta-analysis by Potvin *et al*. [8] reports significant increases in *in vivo* peripheral levels of IL-1RA, sIL-2R, and IL-6 in schizophrenia patients, supporting evidence of immune activation and an inflammatory syndrome in schizophrenia. Studies show that IL-6 mediates the deleterious effects of noncompeting N-methyl-D-aspartate (NMDA) antagonists on cortical interneurons [11] and cytokines have an impact on the regulation of neuroplasticity, cellular resilience, and apoptosis control [12]. TNF-alpha seems to inhibit brain-derived neurotrophic factor (BDNF) release, compromising the protective effect of this neurotrophin.

A number of studies report that treatment with antipsychotic drugs affects the cytokine network [13]. This supports the rationale that the influence of antipsychotics on the cytokine systems may be responsible for their clinical efficacy in schizophrenia. Peripheral immune processes may be mirrored in the brain by microglial cells [6]. The 'microglia hypothesis of schizophrenia' suggests that the release of proinflammatory cytokines, kynurenines, nitric oxide (NO) and reactive oxygen species (ROS) by activated microglia might cause neuronal degeneration, white matter abnormalities and decreased neurogenesis [14–16]. Recent positron emission tomography (PET) studies during acute psychotic episodes in early schizophrenia suggest an activation of microglia [17, 18].

If MXT, which is already on the market for the treatment of heart disease, cancer and rheumatic diseases, proves effective for reducing symptoms of schizophrenia then this could transform the suffering evidenced by patients which severely impairs their quality of life. Targeting the inflammatory response is a major focus of novel drug development especially relevant to the treatment of Treatment Resistant Schizophrenia (TRS). Based on the evidence of strong anti-inflammatory properties of MXT, we propose that low-dose MXT may be an effective therapy in early schizophrenia.

### AIMS

To test the prediction that addition of MXT to treatment as usual (TAU) for patients with early schizophrenia will result in following outcomes:

- ○ To demonstrate that the trial design is acceptable to participants including randomisation to x arms
- ○ To demonstrate that the interventions are acceptable to participants and indicate likely attrition rates and tolerabilityImprovement in negative symptoms
- ○ Improvement in positive symptoms
- ○ Improvement in social functioning
- ○ Improvement in cognitive functions

## Methods/Design

### Overview

This is a double-blind placebo-controlled trial of MXT added to TAU for patients suffering from schizophrenia, schizoaffective disorder, psychosis not otherwise specified or schizophreniform disorder. The ethical approval covering all sites was obtained from the Research and Ethics Committee of the Pakistan Institute of Learning and Living and was submitted to the funding body, SMRI (Project Reference: PILL/SMRI/12627). Patients will be recruited from inpatient and outpatient settings. They will initially be approached by the treating psychiatrists and the multi-disciplinary teams (MDT). Patients will continue to take TAU during the trial. Although, the study assessments will be carried out by the research assistants (RAs), the responsible clinician (consultant psychiatrist) will remain in charge of the overall treatment. Antipsychotic dosage will be kept constant, unless clinically warranted, any changes will be recorded on monthly reviews.

After consenting to take part in the study, 72 patients will be randomised into two groups: 36 in each arm over the 3-month period. Full clinical assessments will be carried out at baseline, 2, 4, 8 and 12 weeks. Social and cognitive assessments will be carried out at baseline and 12 weeks (Table 1). MXT once a week or placebo once a week will be added to TAU. Tolerability will be assessed by self report. During the course of the study the patient will also have additional support by doctors and RAs from the research team and continued support from their mental health care team. If necessary, members of the research team will be available 24 hours a day, 7 days a week.

**Table 1:** **Schedule of assessments**

Assessment	Who			When					
	Patient	Research assistants	RMO team	Screening	Randomisation	Week 2	Week 4	Week 8	Week 12
Case note review			x						x
SCID		x		x					
Drug treatment history		x		x	x	x	x	x	x
Medical history			x	x					
Physical exam		x		x					x
Body weight and BMI		x			x				x
BP and HR		x		x	x	x	x	x	x
Lab screen^a^			x	x		x	x	x	x
Pregnancy screen (urine)		x			x			x	x
Inclusion criteria		x		x					
Exclusion criteria		x		x					
Withdrawal criteria		x			x	x	x	x	
Consent	x	x		x	x				
PANNS		x			x	x	x	x	x
CGI		x			x	x	x	x	x
GAF		x			x				x
QOL		x			x				x
Social function scale		x			x				x
Cognitive assessments		x			x				x
Antipsychotic side-effects		x			x	x	x	x	x
Side-effects									
Spontaneously reported adverse effects and									
methotrexate toxicity check list	x	x			x	x	x	x	x
Compliance monitoring		x				x	x	x	x

^a^Lab screen includes FBC, renal function and liver functions tests. Patients will continue to have these tests at 2-weekly intervals if there are abnormalities on the 6-week blood tests that do not warrant withdrawal from the study. These blood tests are also repeated 4 weeks after finishing the study. Additional blood tests are conducted at baseline and 12 weeks.

*Abbreviations*: *BMI* body mass index, *BP* blood pressure, *CGI* Clinical Global Impression, *FBC* full blood count, *GAF* Global Assessment of Functioning, *HR* heart rate, *PANNS* Positive and Negative Syndrome Scale, *QOL* quality of life, *RMO* resident medical officer, *SCID* Structured Clinical Interview for DSM-IV.

### Allocation

In this study patients will be allocated to treatment group according to a randomised permuted blocks algorithm, after stratification by centre. Allocation will be determined in Manchester by pseudo-random number generation by the trial statistician. The results will be conveyed to the trial pharmacist so that medication can be given to participants without anyone else on the team being aware of the allocation (that is, double-blind).

### Blinding

This is a double-blind, randomised, placebo-controlled feasibility study. Random allocation to treatments will be undertaken by a statistician who will have no knowledge of the patient characteristics. The details of the allocation will be concealed from the research team until all data collection has been completed. Blinding of participants and treating physicians to allocation status will be assured by identical capsule appearance, by identical labelling between placebo and the active drugs (apart from labels identifying the patient). Blinding of the capsules, packaging and labelling, will be undertaken by pharmacist independent of the study. Study investigators and researchers administering the assessment measures will not be aware of the study drug allocated to the individual. In the event a patient develops any side- effects, where drug unblinding is required, the treating physician will be made aware of the study drug, possible side-effects and make the appropriate decision whether to continue or discontinue the drug. The safety and wellbeing of the patient will be paramount at all times.

### Statistical consideration

Our sample size consideration is based on the hypothesis that there will be a significant difference in changes from baseline to end-point on the clinical outcome measures and the cognitive function measures between the MXT group and the TAU group. With 32 participants per group, this study will have 80% power to detect a standardised effect size of 0.53 (medium by the definition of Cohen) by using a 2-sided significant level of 0.20. As this is a preliminary study, we used a less stringent significance level so as not to miss a promising effect rather than being too concerned by false positives. The estimated loss to follow-up rate is 10% and, therefore, a total of 72 patients (36 MXT, 36 TAU) are needed. Our sample size is adequate to estimate the parameters of acceptability and tolerability to the necessary degree of precision.

The planned analyses will be by intention-to-treat (ITT). Careful consideration will be given to the potential biases arising from the drop-out and missing data. Group differences in the change of clinical outcome measures and cognitive function measures at baseline to 12 weeks will be assessed using the *t*-test or analysis of covariance (ANCOVA) adjusting for baseline differences. For repeatedly measured clinical outcomes at baseline, 2, 4, 8 and 12 weeks, we will also use generalised estimating equations (GEE) to estimate the effect of MXT treatment on longitudinal outcomes, including baseline values and time terms as covariates. GEE offers a robust method to take account of the within-subject correlation in longitudinal data.

### Inclusion/Exclusion criteria

#### Inclusion criteria

- Signed informed consent, indicating that the subject understood the purpose of and procedures required for the study, before the initiation of any study specific procedures
- Aged 18 to 35 years
- *Diagnostic and Statistical Manual-IV* (DSM-IV) diagnosed schizophrenia, schizoaffective disorder, psychosis not otherwise specified or schizophreniform disorder
- Early schizophrenia (within first 5 years of diagnosis)
- Competent and willing to give informed consent
- Medication remained stable 4 weeks prior to baseline
- Able to take oral medication and likely to complete the required evaluations
- Female participants of child-bearing capability must be willing to use adequate contraceptives for the duration of the study, and willing to have a pregnancy test pre- treatment and at 10-weekly intervals while on study medication. Adequate contraception is defined as use of contraceptive double barrier system (that is condom and spermicide) or contraceptive implant, oral contraceptive or injected depot contraceptive plus other form of contraceptive, that is a condom. Females will be considered incapable of child-bearing if they are 1 year post-menopausal or irreversibly surgically sterilised

#### Exclusion criteria

- Violation of any inclusion criteria
- Failure to perform screening or baseline examinations
- Relevant *International Statistical Classification of Diseases and Related Health Problems* (ICD-10) organic brain disease or neurological diagnoses (including electrocardiogram (ECG) conduction abnormalities, neurological disorder, or an active seizure)
- Patients with liver disease and polyarthritis
- Patients who will meet the criteria for a DSM-IV-text revision(TR) diagnosis of alcohol or substance abuse (other than for nicotine) within the last month or the criteria for DSM-IV-TR alcohol or substance dependence (other than for nicotine) within the last 6 months
- Any change of psychotropic medications within the previous 4 weeks
- Pregnant or lactating women and those of reproductive age without adequate contraception
- Relevant medical illness will be determined in the first instance by asking the patient's mental health care team if the patient has any medical condition/problems. After consent has been obtained the research nurse/research doctor will then have access to the patients’ notes and will assess patient eligibility to take part in the clinical trial by scrutinising the patients’ past medical history, most recent blood results, ECGs, as well as any physical tests that have been performed on the patient. If there are any deviations from the ‘norm’ the investigators will assess the eligibility of the individual patient. Automatic contraindications will include significantly impaired renal function, significantly impaired hepatic function and pre-existing blood dyscrasias, such as significant marrow hypoplasia, leukopenia, thrombocytopenia or anemia.

### Interval of assessments

There will be a screening, a randomisation and follow-up visits. Full clinical assessments (Positive and Negative Syndrome Scale (PANSS), Clinical Global Impression (CGI), Global Assessment of Functioning (GAF), Social Functioning Scale (SFS) and Quality of Life Scale (QLS)) will be carried out at baseline, 2, 4, 8 and 12 weeks. Social and cognitive assessments (CogState, IQ using Wechsler Adult Intelligence Scale (WAIS) block design, Coughlan Learning Task, Stroop Task, Verbal Fluency) will be carried out at baseline and 12 weeks. The clinical interview and ratings will take approximately 50 minutes. The neuropsychological assessments will take about 60 to 80 minutes. The patients will primarily be seen at their treating team’s base. In special circumstances, if requested by the treating team or patient, the RAs can visit at home.

### RA training and inter-rater reliability

Research assistants in Karachi were trained in Structured Clinical Interview for DSM-IV (SCID), clinical and neuropsychological assessments at the University of Manchester for a previous SMRI-funded study. Inter-rater reliability sessions will be conducted by local principle investigators (PIs) using training videos.

### Treatments

#### Treatment as usual

The patients can be on either first or second generation antipsychotic medications, as deemed suitable by the responsible psychiatrist. From our experience with previous studies there is no major advantage of one generation of drugs over the other.

### Study drug dosage

MXT 7.5 mgs orally once a week) for a 3-month period as an augmentation agent with TAU. All participants will also take folic acid 5 mgs/day orally for 6 days a week (except the day MXT is given), which is routinely given with MXT to prevent vitamin deficiencies.

### Outcome measures for tolerability and acceptability

The definition of tolerability refers to adverse events. An adverse event is the development of an undesirable medical condition or the deterioration of a pre-existing medical condition following or during exposure to a pharmaceutical product, whether or not considered causally related to the product.

At each clinical assessment (baseline, 2, 4, 8 and 12 weeks and early exit from the study) we will collect spontaneously reported adverse effects and also administer an adverse effect check list covering 8 key symptoms/signs of MXT toxicity (rash, oral ulceration, nausea and vomiting, diarrhea, new or increasing dyspnea, new or increasing dry cough, severe sore throat, abnormal bruising). If any of these eight symptoms are present MXT will be withheld until the case has been discussed with the onsite investigator. In addition, severe sore throat or abnormal bruising will lead to an urgent full blood count (FBC) being conducted, the result of which will be considered by the investigator in reaching a decision on the patient continuing in the trial. The routine blood tests at baseline, 2, 4, 6 and 12 weeks will be reviewed by the site investigator within 4 days of being taken and the patient withdrawn from the study if any indices give cause for concern (that is are outside pre-specified parameters).

Acceptability is defined as treatment discontinuation in terms of the number of patients who terminated the study early (drop-outs) for any reason following randomisation. We will request an exit interview with all participants who did not complete the study to explore tolerability and acceptability.

### Clinical outcome measures

1. Negative symptom severity as defined by negative syndrome subscale score on the PANSS and/or Negative Symptom Assessment Scale;
2. Full PANSS and positive syndrome subscale score [19]
3. CGI
4. Functional outcome:
  GAF from DSM-IV (2000) [20]
  SFS self-rating in seven domains [21]
  QLS for treatment effects related to deficit or negative symptoms [22]

#### Cognitive function measures

We will use pencil and paper tests and CogState; measuring all seven domains recommended by MATRICS (NIMH initiative). These domains include speed processing, attention/vigilance, working memory (nonverbal and verbal), verbal learning, visual learning, reasoning and problem solving and social cognitions. We will use the following tests:

1. IQ: using WAIS block design
2. Stroop Task, for divided attention and processing speed
3. Coughlan Learning Task (Verbal and Visual)
4. Verbal Fluency (words and categories)

### Side-effects

Self reported antipsychotic side-effects: Antipsychotic Non-Neurological Side-Effects Rating Scale (ANNSERS) [23].

### Safety assessments

#### Medical history

The primary purpose of screening is to check for inclusion and exclusion criteria. The RAs will arrange for a doctor to complete checklists for medical history, review of systems and physical exam. These assessments will be repeated after 3 months in the trial or at the time of withdrawal from the study.

### Physical assessments

ECG, blood pressure, pulse, height, waist, weight and body mass index (BMI).

### Blood investigations

FBC, erythrocyte sedimentation rate, LFTs, lipid profile, urea, electrolytes, cytokines and C-reactive protein.

Patients will have FBC and renal and LFTs before starting treatment and repeated at 2, 4, 6 and 12 weeks. Remaining investigations will be at baseline and 12 weeks. Patients will be advised to report all symptoms and signs suggestive of toxicity and infection, especially sore throat. Patients will continue to have a 2-weekly FBC after 6 weeks if there are abnormalities on the 6-week FBC that do not warrant withdrawal from the study. For patient safety, full blood monitoring will be repeated at 4 weeks after finishing the study, at week 16.

### Additional safety measures

1. At each clinical assessment (baseline, 2, 4, 8 and 12 weeks and early exit from the study) we will collect spontaneously reported adverse effects and also administer an adverse effect check list covering 8 key symptoms/signs of MXT toxicity (rash, oral ulceration, nausea and vomiting, diarrhea, new or increasing dyspnea, new or increasing dry cough, severe sore throat, abnormal bruising). If any of these eight symptoms are present MXT will be withheld until the case has been discussed with the onsite investigator. In addition, severe sore throat or abnormal bruising will lead to an urgent FBC being conducted the result of which will be considered by the investigator in reaching a decision on the patient continuing in the trial.
2. The routine blood tests at baseline, 2, 4, 6 and 12 weeks will be reviewed by the site investigator within 4 days of being taken and the patient withdrawn from the study if any indices give cause for concern (that is are outside pre-specified parameters).
3. Our administration of a symptoms checklist and monitoring of FBC, LFTs and renal function is similar to monitoring advised in the UK (http://cks.nice.org.uk/dmards#!scenario:8).
4. Folic acid, 5 mg once weekly will be given, not on the same day as MXT, preferably the day after [24]. Folic acid reduces toxic effects of MXT and improves continuation of therapy and compliance [25, 26]. There is also evidence that increasing folate levels improves outcomes in schizophrenia [27].
5. Given that participating psychiatrists will have little or no experience in prescribing MXT, we will ensure that there is an on-call system such that all investigators can get immediate expert advice from a physician in Pakistan who is experienced in prescribing and monitoring methotrexate.
6. MXT will be dispensed 2-weekly throughout the study to limit the supply participants have, facilitate monitoring and adherence. The label will state: ‘Take once a week’.
7. Participants will all have an information leaflet with safety information.
8. The patients will have access to their RA at all times, who will be able to contact the research team medical staff 24 hours a day, 7 days a week for support and advice.

### Study procedures

#### Recruitment

Responsible consultant psychiatrists will be approached and asked if they will allow their patients to take part in this research study. The research clinician, initially, will approach the clinical teams to inform them about the research study, specifically with regard to inclusion and exclusion criteria. The research clinician will establish a good working relationship with individual clinical teams. With regular contact, either by phone or by visits, patients who are suitable to take part in the research study will be identified in collaboration with the treating teams. The consultant psychiatrist will then introduce the study to the patient if they meet the inclusion criteria, are clinically stable, and the MDT agree the patient could be a possible participant. With the patient’s consent, the research clinician will visit them and explain the research study verbally as well as provide them with the written information. At least 24 hours will be given to the patient to allow time to read and understand the patient information sheet. If they are willing to take part, a meeting will be organised with the patient in order to obtain consent for the research and also consent for the research team to have access to their medical notes.

#### Screening visit

At the screening visit a confirmation of a patient’s suitability for the trial will be carried out. The patient will be assessed against the inclusion/exclusion criteria, have a confirmation of their diagnosis, confirmation of consent to take part in the trial and pregnancy testing if appropriate. The RAs will arrange for a doctor to complete a checklist for medical history, physical examination and hematological investigations.

#### Randomisation visit

The baseline clinical and neuropsychological measures will be completed and the patient will be randomised to one of the two arms, allocated a unique identification number and will commence study drug treatment.

The patient will be given a study information card explaining that they are in a clinical study and are taking MXT or placebo; the telephone numbers of the senior research clinicians, the clinical trial office and the name of the local PIs will be provided.

In accordance with (International Conference for Harmonisation) Good Clinical Practice ((ICH) GCP) (1996) [28], copies of all the above will be placed in the patient’s medical notes along with contact names and telephone numbers.

#### Follow-up visits

The first follow-up visit will be after 2 weeks of the randomisation to complete clinical assessments. There will be subsequent visits at week 4, 8 and 12. The study drugs will be dispensed from the local pharmacy departments. The RAs will collect and deliver these to the patient at a previously agreed upon location. This will give the opportunity for the research team to closely monitor the patient’s physical and mental health, side-effects and compliance.

Female patients of childbearing capacity taking part in the clinical trial will have a pregnancy test at baseline, 8 and 12 weeks. If they should become pregnant during the course of the study, they will be withdrawn from the study.

During the course of the study the patient’s individual consultant and mental health care team will be responsible for their overall care. If any concerns do occur with regard to the research study, the research team will be contactable to discuss these concerns 24 hours a day, 7 days a week.

#### Final visit

At this visit all clinical, social, cognitive, physical and hematological assessments will be completed. Trial medication will cease.

At all visits the patient’s cumulative clinical drug treatment will be updated from the case notes.

#### Study coordination

Weekly meetings by local investigators will occur to coordinate the study. The chief investigator will conduct 2-weekly tele- and video-conference meeting. The access grid facility at the University of Manchester will also be available.

#### Patient safety

Day-to-day care will remain the responsibility of the consultant in charge of the patient. However, study related safety concerns will be the responsibility of the local PI. The PIs and the co-investigators will be contactable at any time via the clinical trials office or/and through the senior research clinicians.

#### Patient withdrawal

Patients may be withdrawn from the treatment study for the following reasons:

1. At their own request.
2. At the discretion of the investigator.
3. If any patients suffers a serious adverse event, or moderate to severe adverse drug reaction.
4. Any patients who meet the criteria for insufficient compliance. This is defined as either taking less than 75% of antipsychotic or trial medication between assessment points at baseline, 6 weeks and 12 weeks; or missing trial medications or antipsychotic medication altogether for 7 days or more at any period.
5. If a patient becomes pregnant.

#### Ethical and regulatory standards

##### Local Research Ethics Committee (LREC)

Ethical approval was obtained from the research and ethics committee of the Pakistan Institute of Learning and Living and was submitted to the funding body, Stanley Medical Research Institute.

##### Data and Safety Monitoring Board (DSMB)

The DSMB will be constituted according to NIH guidelines, for oversight and monitoring of the conduct of clinical trial. DSMB will meet every 6 months to ensure the safety of participants and the validity and integrity of the data.

##### Declaration of Helsinki

The research study will be performed in accordance with the guidelines in the Declaration of Helsinki (1974) as revised in Tokyo (1975), Venice (1983), Hong Kong (1989), South Africa (1996) and Scotland (2000).

All members of the research team will comply with ICH/GCP Guidelines (1996), which are consistent with principles that have their origin in the Declaration of Helsinki.

##### Team expertise

Five of the investigators have extensive experience in treatment and management of schizophrenia (Chaudhry, Haddad, Husain, Kazmi, Rahman and Deakin). Kazmi and Rahman as local investigators will be important components of success in recruitment. All investigators have been closely involved in setting up the methodology and software for gathering clinical and research data in patients with psychosis. Chaudhry and Husain will oversee the training of RAs in recruiting patients and in diagnostic and symptom ratings. All of the investigators were involved in feasibility study for this trial. Deakin, Haddad and Chaudhry are experts in psychopharmacology. Chaudhry and Husain have been PIs in a number of multicentre trials funded by the Medical Research Council (MRC) and SMRI in schizophrenia and have much experience of large-scale treatment studies in psychosis. Chaudhry and Husain have published longitudinal studies on negative symptoms, cognition and quality of life. Husain, Chaudhry, Kazmi, Rahman and colleagues ran the SMRI trial of minocycline.

## Discussion

Evidence suggests inflammatory processes are involved in the pathogenesis of schizophrenia and anti-inflammatory treatments have shown to have some beneficial effects. MXT is a known immuno-suppressant and anti-inflammatory drug. The aim of the this study is to evaluate the effectiveness of MXT added to TAU on positive and negative symptoms, cognitive and social functioning and quality of life of patients suffering from schizophrenia.

## Trial status

This clinical trial was registered in February of 2014. The study is currently recruiting participants. The estimated study completion date is December 2015. Please refer to this study by its ClinicalTrials.gov identifier: NCT02074319.

## Abbreviations

ANCOVA: analysis of covariance
ANNSERS: Antipsychotic Non-Neurological Side-Effects Rating Scale
BDNF: brain-derived neurotrophic factor
BMI: body Mass Index
BP: blood Pressure
CGI: Clinical Global Impression
CRP: C-reactive protein
DHFR: dihydrofolate reductase
DSMB: Data and Safety Monitoring Board
DSM-IV-TR: *Diagnostic and Statistical Manual-IV-text revision*
ECG: electrocardiogram
FBC: full blood count
GAF: Global Assessment of Functioning
GCP: Good Clinical Practice
GEE: generalised estimating equations
ICD-10: *International Statistical Classification of Diseases and Related Health Problems*
ICH: International Conference for Harmonisation
IFN-gamma: interferon-gamma
IL: interleukin
IL-1RA: interleukin 1 receptor antagonist
IQ: Intelligence Quotient, ITT, intention-to-treat
LFTs: liver function tests
LREC: Local Research Ethics Committee
MATRICS: Measurement and Treatment Research to Improve Cognition in Schizophrenia
MDT: multi-disciplinary teams
MRC: Medical Research Council
MXT: methotrexate
NMDA: N-methyl-D-aspartate
NO: nitric oxide
PANSS: Positive and Negative Syndrome Scale
PET: positron emission tomography
PI: principal investigator
QLS: Quality of Life Scale
QOL: quality of life
RA: research assistants
RMO: resident medical officer
ROS: reactive oxygen species
SFS: Social Functioning Scale
SCID: Structured Clinical Interview for DSM-IV
sIL-2R: soluble IL-2 receptor
SMRI: Stanley Medical Research Institute
TAU: treatment as usual
TNF-alpha: tumour necrosis factor-alpha
TRS: Treatment Resistant Schizophrenia
UK: United Kingdom
WAIS: Wechsler Adult Intelligence Scale.
